# Radical prostatectomy in metastatic prostate cancer: is there enough evidence? | *Opinion: Yes*

**DOI:** 10.1590/S1677-5538.IBJU.2016.05.04

**Published:** 2016

**Authors:** Walter Henriques da Costa, Gustavo Cardoso Guimarães

**Affiliations:** 1 AC Camargo Cancer Center, SP, Brasil

**Keywords:** Prostatectomy, Prostate, Prostatic Neoplasms

Prostate cancer (PCa) is the most frequent and the second ranked cause of cancer deaths among men each year. The vast majority of patients are diagnosed with localized disease, however it is estimated that 35,000 American men were diagnosed with locally advanced or metastatic prostate cancer (mPCa) in 2015 (1). During the last few years we have seen notable advances in the treatment of mPCa with the introduction of several second-line hormonal therapy options, immunotherapy and cytotoxic chemotherapy in hormone sensitive disease (2). With newer therapies that prolong survival in patients relapsing with mPCa and the increasingly widespread use of prostate-specific antigen (PSA) testing, men with metastatic disease might have lower disease burden at diagnosis than in the past decades (3). Although recent data suggest a relative improvement in 2-year overall survival in mPCa patients treated with systemic therapy, the long-term survival still remains disappointing. Actually, patients with mPCa and non-metastatic PCa present 5-year cancer-specific survival (CSS) rates of 28% and 99%, respectively (4). Thus, there is clearly room for improvement in the treatment of mPCa patients.

The concept of cytoreductive surgery is well established in a number of tumors such as ovarian (5) and kidney cancer (6). Cytoreduction is still a largely unexplored subject in PCa, however recent evidences have shown its importance in the mPCa scenario. Possible theoretical advantages of cytoreductive prostatectomy (CP) include primary tumor debulking and improved response to systemic therapies. Kadmon et al. described one of the first studies addressing this issue in 1982. In an animal model, the authors used a PCa cell line that uniformly resulted in metastatic lung colonies. The mice were treated with either single-dose chemotherapy, surgical excision of the primary tumor, or a combination of tumor excision and postoperative single-dose chemotherapy. Tumor excision followed by postoperative chemotherapy resulted in a decrease in the number of metastatic sites in the lungs and prolonged survival. Of the combined group, 42% were long-term survivors (tumor-free >180 days), which was not seen in the non-surgery group (7). One additional potential mechanism of action for CP is based on the concept of tumor self-seeding. It is known that circulating tumor cells (CTCs) have the potential to seed metastases in distant organs in a unidirectional process. CTCs can also seed and then colonize their own tumors of origin, which accelerates tumor growth, angiogenesis and stromal recruitment through seed-derived factors (8). CTCs detected by the CellSearch^®^ assay were shown to be prognostic for survival in patients with metastatic castration-resistant prostate cancer (CRPC). de Bono et al. found worse overall survival for patients who had ≥ 5 CTCs compared to those who had <5 CTCs prior to starting a new cytotoxic therapy. Patients who had ≥ 5 CTCs had a median overall survival of 11.5 months, compared to 21.7 months for those who had <5 CTCs (HR 3.3, 95 % CI 2.2 – 5.1, p value <0.0001) (9). Tzelepi et al. analyzed the outcome of 40 PCa patients with either locally advanced or lymph node metastases that were treated with 1 year of androgen deprivation therapy (ADT) and docetaxel chemotherapy previously to radical prostatectomy (RP) and pelvic lymphadenectomy. Despite the extensive pretreatment, all RP specimens contained vital cancer cells with potentially lethal properties. The authors suggest that persisting intraprostatic cancer foci might be involved in local progression and development of distant metastases (10). Therefore, it is feasible that the primary tumor removal would reduce overall tumor volume, which may limit the establishment of new metastatic sites and allow other therapies to work more effectively.

A series of clinical studies already indicated a beneficial role of cytoreductive surgery in mPCa patients. In a secondary analysis of the SWOG 8894 trial, Thompson et al. randomized 1,286 men with metastatic prostate cancer to orchiectomy and placebo or orchiectomy and flutamide. One hundred and forty-eight patients had previously undergone RP for what was believed to be localized PCa. The impact of previous local therapy to the prostate on clinical outcomes was investigated. Previous RP in patients with mPCa was associated with a statistically significant decrease in the risk of death (HR=0.77, 95% CI, 0.53–0.89) when compared to those who did not undergo earlier prostatectomy (11). The potential of cytoreductive transurethral resection of the prostate (TURP) was also evaluated in 146 men with hormone sensitive prostate cancer. All patients received complete androgen blockade (CAB) as initial systematic therapy. Patients were divided in TURP + CAB vs. CAB only groups. Clinical and pathologic characteristics were comparable between the two groups. Patients who underwent a TURP had lower PSA nadir (median 0.15 ng/ ml vs. 0.82 ng/ml, P = 0.015) and longer time to PSA nadir (11.2 months vs. 6.4 months, P < 0.001). More patients in the non-TURP group developed hormone refractory PCa (P = 0.007) (12).

It is known that 40% of patients with PCa palliated with ADT alone (i.e. without curative intent) will need lower urinary tract procedures and 10% will need upper urinary tract procedures before death. Of those without metastatic disease, 60% will require lower and 20% upper urinary tract procedures with a mean survival of 94 months (13). Complications include subvesical obstruction, recurrent gross hematuria, upper urinary tract dilatation, rectourethral or rectovesical fistulae and rectal obstruction. One of the main issues regarding local therapy of the primary tumor is that it may possibly prevent or delay the onset of clinical symptoms from local progression. According to retrospective data for occult lymph nodal disease, the incidence of symptomatic disease progression requiring palliative surgical procedures is lower in patients who undergo initial RP than in those treated with systemic therapy alone. Wiegand et al. performed a retrospective analysis in 192 lymph node positive (pN+) PCa patients. Patients were divided into three groups: RP alone, ADT alone or RP + ADT. The incidence of local relapse in the three treatment groups (RP, ADT and RP + ADT) was 40.2%, 59.5% and 12.9%, respectively. Logistic regression analysis of predictors for symptomatic local relapse indicated that ADT alone (OR = 8.67, P < 0.001) had significantly higher odds of symptomatic local relapse compared with RP alone. The findings suggest that RP with or without ADT leads to improved outcomes in patients with pN+ prostate cancer (14). A later study analyzed the impact of primary tumor treatment in 263 metastatic CRPC patients. Eligible patients were men who had progressive disease (defined by a rising PSA level or the presence of new metastases) despite treatment with ADT and with castrate levels of testosterone. Men were divided into three groups. Group 1 received previous local treatment of primary prostate cancer by RP (n = 45) with or without postoperative radiotherapy; group 2 received definitive external beam radiotherapy (EBRT; n = 45); and group 3 received no initial local prostate therapy (Nil; n = 173) (these patients were treated with watchful waiting or ADT). The most common local complications were bladder outlet obstruction (35.0%) and ureteric obstruction (15.2%). Treatment of the prostate by either RP or EBRT (groups 1 and 2) significantly reduced the incidence of subsequent local complications compared to that in patients who had no primary treatment (group 3) (32.6% vs. 54.6%; P = 0.001). The estimated relative risk of local complication among men who had no local prostatic treatment compared to that in patients receiving local treatment was 1.68 (95% CI, 1.21–2.33). In addition, RP only patients showed a significantly lower probability of local complications compared to EBRT only patients (20.0% vs. 46.7%; P = 0.007). The analysis supports the hypothesis that primary local prostatic treatment provides a palliative benefit to men who later develop CRPC (15).

A central question is whether the characteristics and the volume of metastatic disease influence the outcome of patients undergoing CP. Which patients would be ideal candidates for the realization of CP? Some recent studies have addressed this issue. In a SEER Database analysis, Culp et al. identified 8185 mPCa patients treated with RP, brachytherapy (BT) or no surgery or radiation (NSR). The 5 years overall survival (OS) was significantly higher in patients undergoing either RP (67.4%; 95% CI, 58.7–74.7) or BT (52.6%; 95% CI, 39.8–63.9) compared with NSR patients (22.5%; 95% CI, 21.1–23.9) (p < 0.001). In addition, undergoing RP or BT was each independently associated with decreased cancer-specific mortality (CSM), with predicted 5 years CSS probabilities of 75.8% and 61.3%, respectively, compared with 48.7% for NSR patients. To determine if the burden of metastatic disease influenced survival among groups, subset analyses were performed based on TNM / AJCC M stage (M1a–c). Compared with NSR patients, men undergoing RP presented decreased CSM regardless of M stage and a higher OS in M1b and M1c disease. Although the baseline expectation would be that the greatest benefit would be seen only in patients with M1a disease, an improved survival was also noted in patients with either M1b or M1c disease, with the most pronounced advantage in patients with M1c disease (16). This finding is extremely relevant because it goes against the concept in which the patient with the greatest potential benefit with CP was precisely the one with minimal metastatic disease. In a later prospective case-control study, Heidenreich et al. explored the role of CP in 23 PCa patients with low volume skeletal metastases (3 or fewer hot spots on bone scan), absence of visceral or extensive lymph node metastases and PSA decrease to less than 1.0 ng/ml after neoadjuvant bicalutamide followed by ADT with LHRH analogues for 6 months (group 1). Patients were compared to a matched control group of 38 men initially treated with ADT only and followed until progression, development of castration resistant PCa or death (group 2). In the CP group, 13 (56.5%) and 4 (14.3%) patients had lymph node metastases and positive surgical margins, respectively. No Clavien grade 4 or 5 complications occurred and 21/23 patients (91.3%) were continent with the use of 0 to 1 pads per day after a median follow-up of 34.5 months. It is important to note that functional outcomes of CP did not differ from the reported outcomes of RP in high risk PCa in terms of postoperative continence recovery. Median time to CRPC was 40 months and 29 months in groups 1 and 2, respectively (p=0.04). Patients in group 1 experienced significantly better clinical progression-free survival (38.6 vs. 26.5 months, p=0.032) and CSS rates (95.6% vs. 84.2%, p=0.043) (17). Finally, a multi-institutional study described peri-operative outcomes and short-term survival analysis of 106 mPCa patients submitted to open or robotic CP. Continence at 90 d after surgery was recorded in 59/106 (55.7%) patients, and most (38/59; 64.4%) were dry, with only 18.6% (11/59) suffering moderate/severe incontinence. The overall complication rate was 20.8%. At the end of the study, 12/106 (11.3%) men died from prostate cancer at a median follow-up of 22.8 months. In this study, men with nonregional nodal metastases had better survival rates after radical prostatectomy than those with skeletal disease (18). Taking into consideration such recent studies, it is concluded that CP is feasible and safe; presents acceptable functional results and it might be an interesting option in the multimodality management of mPCa.

Based on the observations and findings listed above, a number of clinical trials have been proposed in order to better define the role of primary tumor treatment in mPCa patients. The UK STAMPEDE (www.clinicaltrials.gov NCT00268476) and Dutch HORRAD (www.trialregister.nl NTR271) trials are evaluating the role of radical intervention in mPCa using radiation as the modality of choice (18). Another recruiting trial is giving patients with metastatic disease who respond to systemic therapy the choice of surgery versus radiation as local therapy (www.clinicaltrials.gov NCT01751438).

So we got the central question: “Radical prostatectomy in metastatic prostate cancer: is there enough evidence?” In our opinion, yes there is enough evidence. At least until the preliminary results of such prospective studies are available, the indication of CP is based on the evidence described above. CP for men with locally resectable mPCa is feasible and safe in expert hands for meticulously selected patients.
